# Burning question: Are there sustainable strategies to prevent microbial metal corrosion?

**DOI:** 10.1111/1751-7915.14347

**Published:** 2023-10-05

**Authors:** Di Wang, Enze Zhou, Dake Xu, Derek R. Lovley

**Affiliations:** ^1^ Electrobiomaterials Institute, Key Laboratory for Anisotropy and Texture of Materials (Ministry of Education) Northeastern University Shenyang China; ^2^ Shenyang National Laboratory for Materials Science Northeastern University Shenyang China; ^3^ Department of Microbiology University of Massachusetts Amherst Massachusetts USA

## Abstract

The global economic burden of microbial corrosion of metals is enormous. Microbial corrosion of iron‐containing metals is most extensive under anaerobic conditions. Microbes form biofilms on metal surfaces and can directly extract electrons derived from the oxidation of Fe^0^ to Fe^2+^ to support anaerobic respiration. H_2_ generated from abiotic Fe^0^ oxidation also serves as an electron donor for anaerobic respiratory microbes. Microbial metabolites accelerate this abiotic Fe^0^ oxidation. Traditional strategies for curbing microbial metal corrosion include cathodic protection, scrapping, a diversity of biocides, alloys that form protective layers or release toxic metal ions, and polymer coatings. However, these approaches are typically expensive and/or of limited applicability and not environmentally friendly. Biotechnology may provide more effective and sustainable solutions. Biocides produced with microbes can be less toxic to eukaryotes, expanding the environments for potential application. Microbially produced surfactants can diminish biofilm formation by corrosive microbes, as can quorum‐sensing inhibitors. Amendments of phages or predatory bacteria have been successful in attacking corrosive microbes in laboratory studies. Poorly corrosive microbes can form biofilms and/or deposit extracellular polysaccharides and minerals that protect the metal surface from corrosive microbes and their metabolites. Nitrate amendments permit nitrate reducers to outcompete highly corrosive sulphate‐reducing microbes, reducing corrosion. Investigation of all these more sustainable corrosion mitigation strategies is in its infancy. More study, especially under environmentally relevant conditions, including diverse microbial communities, is warranted.

## Graphical abstract

Microbial corrosion of metals is a major global economic burden. This manuscript briefly summarizes traditional strategies for corrosion mitigation and describes novel, more sustainable, approaches that show promise but require further experimental evaluation

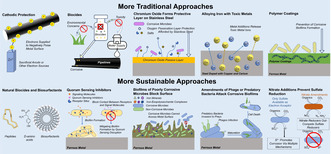

## INTRODUCTION

Microbial corrosion of metals is an expensive, insidious process that negatively impacts UN sustainability goals for sustainable infrastructure, energy, water and sanitation, and health (Knisz et al., 2023; Little et al., 2020; Xu et al., 2023). It weakens structures, causes pipeline leaks, and can destroy metal‐containing medical devices (Wade, 2022; Xu et al., 2023). In addition to direct costs estimated to exceed a trillion dollars a year, corrosion costs also include additional, difficult‐to‐assess environmental and safety issues, including substantial carbon emissions for the manufacture of replacement steel (Cámara et al., 2022; Iannuzzi & Frankel, 2022). As detailed below, current practices for mitigating microbial metal corrosion require substantial energy inputs and/or toxic chemicals. Furthermore, their effectiveness is limited. Alternative chemical/metallurgical strategies that have been proposed are too expensive for widespread application or rely on the release of toxins that are not environmentally acceptable. Therefore, a burning question for microbial biotechnology is: Can a sustainable biotechnological strategy be developed to prevent microbial metal corrosion?

## How do microorganisms corrode metal?

**FIGURE 1:**
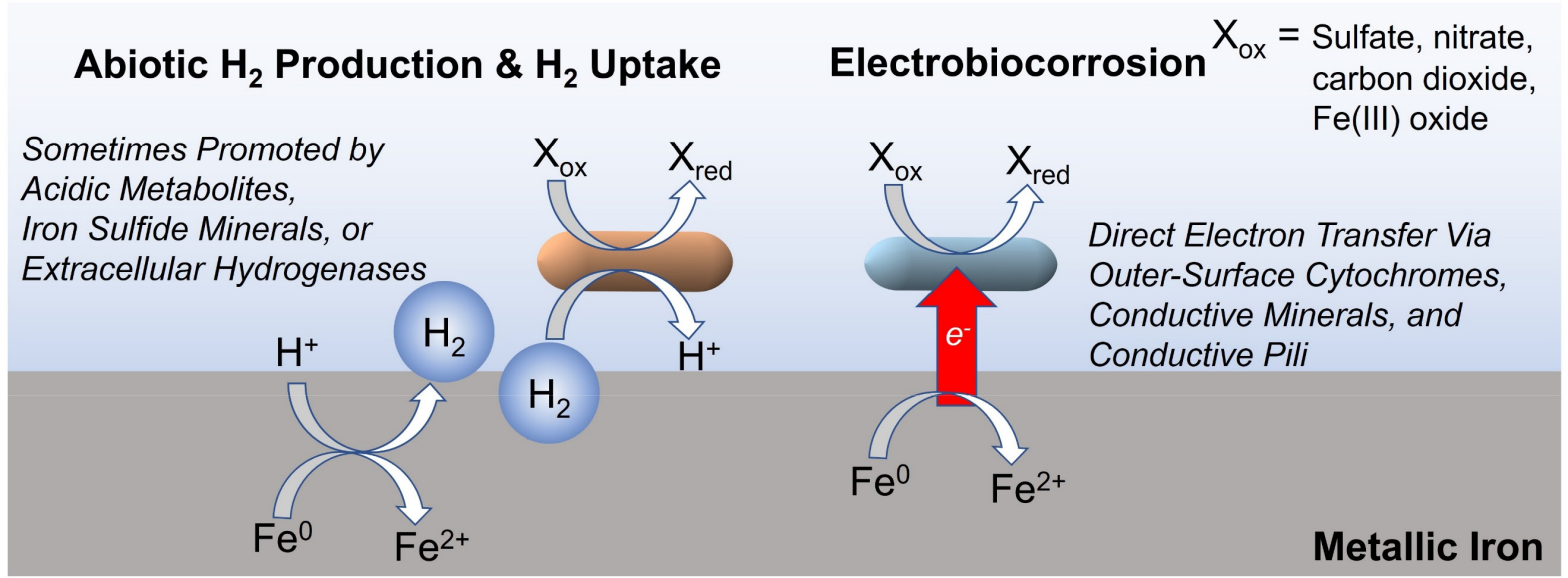
Primary mechanisms for microbial corrosion of ferrous metals under anaerobic conditions.

The rational development of strategies to prevent microbial metal corrosion requires an understanding of microbial corrosion mechanisms. Microbial corrosion studies have primarily focused on the corrosion of iron‐containing (ferrous) metals because ferrous metals are the most common material in metal structures and pipelines, and they are highly susceptible to corrosion. Multiple microbial mechanisms for ferrous metal corrosion are known (Figure 1). Detailed reviews of this topic are available (Wade, 2022; Xu et al., 2023). Briefly, all mechanisms involve the oxidation of metallic iron (Fe^0^) to Fe^2+^:

(1) $${\mathrm{Fe}}^{0}\to {\mathrm{Fe}}^{2+}+2\;e^{-}.$$

This half reaction can only proceed in conjunction with the reduction of a molecule that can accept the electrons released from Fe^0^. In air, O_2_ can accept the electrons in abiotic reactions that not only generate Fe^2+^ but also further oxidize Fe^2+^ to Fe^3+^ with the formation of the iron oxyhydroxides commonly known as rust. This aerobic oxidation is generally self‐limiting, as rust builds up and precludes O_2_ further access to the Fe^0^ surface.

It is under anaerobic conditions that microbes have their biggest corrosive impact. Electrons released from Fe^0^ (reaction 1) can abiotically react with protons to produce H_2_:

(2) $$2\;H^{+}+2\;e^{-}\to H_{2}.$$

Microbes can accelerate this reaction by generating acidic metabolites or by consuming H_2_ with the reduction of common electron acceptors for anaerobic respiration such as nitrate, Fe(III), sulphate, or carbon dioxide (Xu et al., 2023). Sulphide produced from microbial sulphate reduction can further accelerate Fe^0^ oxidation in a reaction with Fe^0^ (Enning & Garrelfs, 2014):

(3) $${\mathrm{Fe}}^{0}+H_{2}S\to \mathrm{FeS}+H_{2}.$$

and iron sulphide precipitates can promote electron transfer between Fe^0^ and H^+^ (reaction 2) (Little et al., 2020).

Alternatively, microbes can forgo H_2_ as an intermediary electron carrier and extract electrons from Fe^0^ through outer‐surface electrical contacts. This ‘electrobiocorrosion’ (Xu et al., 2023) takes place at the metal‐microbe interface through outer‐surface electrical contacts such as *c*‐type cytochromes, conductive minerals, and electrically conductive pili (Hernández‐Santana et al., 2022; Holmes et al., 2022; Jin et al., 2023; Tang et al., 2019; Tang et al., 2021; Zhou, Li, et al., 2022). Cells at distance from the metal surface may also feed on Fe^0^ via long‐range electron transfer through conductive biofilms (Jin et al., 2023).

At present, there is a lack of understanding of the relative importance of the known mechanisms for microbial iron corrosion under different conditions. However, a commonality among all the known mechanisms is the importance of biofilm formation. Electrobiocorrosion requires microbial contact with the metal surface. Also, microorganisms need to be in close association with the metal surface to promote the oxidation of Fe^0^ coupled to H_2_ production (reactions 2 and 3) via metabolite production and to compete for the H_2_ released. Therefore, sustainable processes for preventing microbial iron corrosion must focus on preventing deleterious corrosive microbes from accessing the metal surface and/or inhibiting their growth and activity.

## What are the current methods for preventing corrosive biofilm growth?

**FIGURE 2:**
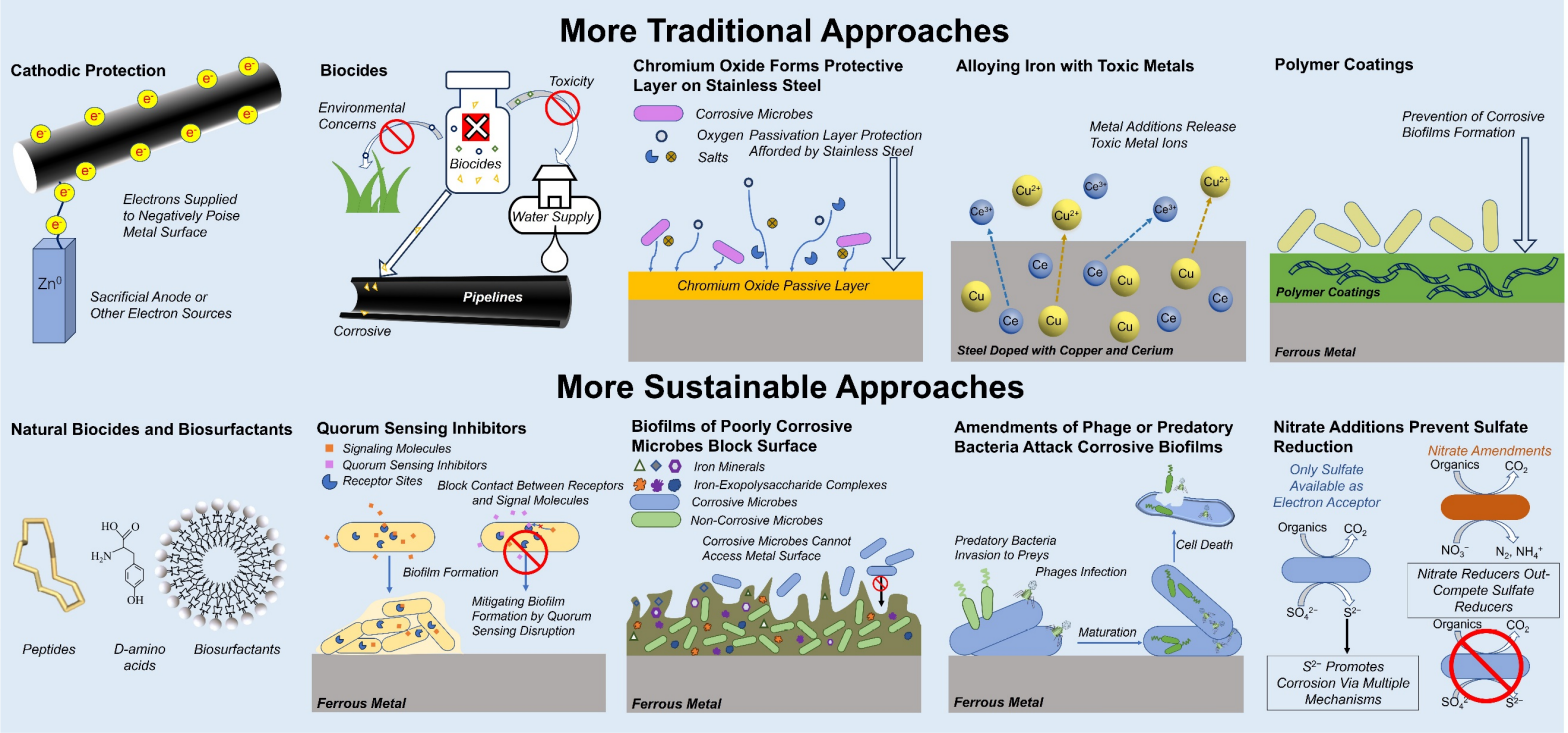
Diversity of strategies that have been proposed mitigating the corrosion of ferrous metals. Details are provided in the main text.

There is a diversity of practices for mitigating microbial metal corrosion (Figure 2). As detailed below, several are limited in the range of environments and structures/devices for which they are practical. Many approaches are not cost effective for broad range application. Often there is little experimental data available to objectively evaluate effectiveness.

### Cathodic protection

A commonly applied approach for corrosion mitigation is ‘cathodic protection’ in which electrons are supplied to the surface of metal pipelines and structures buried in soil or in marine environments to poise the metal at a negative potential. However, there is a lack of rigorous experimental evidence for the effectiveness of cathodic protection in preventing microbial iron corrosion. In fact, in some instances, the cathodic protection approach can provide electrons for electroactive microorganisms, stimulating microbial corrosive activity (Liduino et al., 2021; Lv & Du, 2018; Thompson et al., 2022).

### Biocides

Periodic scraping of the metal surface (i.e., pigging) and/or application of biocides are traditional treatments for corrosive biofilms within oil pipelines and cooling water systems (Jia, Li, et al., 2017; Keasler et al., 2017). Commercial biocides can be divided into oxidizing and non‐oxidizing biocides (Jia, Unsal, et al., 2019; Lekbach et al., 2021). Oxidizing biocides such as chlorine, bromide, and hydrogen peroxide have the limitation that, in addition to destroying microbes, they can also corrode metals (Zhong et al., 2020). Non‐oxidizing biocides like THPS (tetrakis hydroxymethyl phosphonium sulphate), glutaraldehyde, ADBAC (alkyldimethylbenzylammonium chloride), and benzalkonium chloride have broad‐spectrum efficacy in killing microbes and are biodegradable (Liu et al., 2017; Okoro, 2015). Abundant reducing agents common in anaerobic environments limit the lifetime of oxidizing biocides, and thus non‐oxidizing biocides typically have longer term biocidal effects (Keasler et al., 2017). However, oxidizing biocides are cheaper. Based on the data from chemanalyst.com, glutaraldehyde, a common non‐oxidizing biocide, costs 2100 USD per tonne in North America in 2023. The price of liquid chlorine per tonne is about a third the price. Thus, both effectiveness and cost need to be considered when choosing a biocide (Videla, 2002).

A drawback to biocides is that they are typically toxic to life forms other than microbes. Thus, it is not feasible to add biocides to water supply or sanitation system infrastructure or for the protection of the outer surface of buried structures, pipelines, or implanted medical devices. Furthermore, even in the limited environments in which biofilm scrapping and biocide treatment are feasible, these approaches are only temporary fixes and need to be repeatedly applied at substantial cost. More sustainable approaches are desired.

### Iron alloys and non‐ferrous metals

Iron can be combined with other metals to fabricate alloys that are more resistant to microbial corrosion. The best known is stainless steel, which contains at least 10.5% chromium. The chromium near the surface of the stainless steel is oxidized in air, forming a layer of chromium oxide that protects the underlying iron from direct oxygen contact in aerobic environments, as well as blocking protons and microbial metabolites that favour the oxidation of Fe^0^ with the production of H_2_ under anaerobic conditions (Ejileugha et al., 2021; Enning & Garrelfs, 2014). Electroactive microbes that can directly extract electrons from metal surfaces can corrode stainless steel, but microbes that rely on H_2_ to shuttle electrons between Fe^0^ and the microbe appear to be ineffective in the corrosion of high‐quality stainless steels with a high chromium content (Holmes et al., 2023; Liang et al., 2021; Tang et al., 2021; Zhou, Li, et al., 2022). The limitation for employing stainless steel in most pipelines and large structures is its four‐fold higher cost compared with more common structural iron forms like carbon steel. Carbon steel contains small amounts of carbon, which improves iron's strength, ductility, and hardness (Dwivedi et al., 2017). Although it is an excellent structural material, it lacks the protective coating that forms on stainless steel, and thus carbon steel is much more susceptible to chemical and microbial attack. For example, like pure Fe^0^ (Tang et al., 2019), carbon steel abiotically produces H_2_ (Hernández‐Santana et al., 2022), whereas high‐quality stainless steel does not (Tang et al., 2021).

Steel doped with copper can leach copper ions, which are toxic to microbes (Zhang et al., 2021). In laboratory studies, the inclusion of ca. 1 wt % copper in steel clearly inhibited corrosive microbes compared to steel without copper (Li, Shi, et al., 2022; Shi et al., 2018; Zeng et al., 2022). Higher copper concentrations diminish steel's toughness, limiting applications (Shi et al., 2018). Copper concentrations below 1 wt % provide negligible antimicrobial efficacy (Fan et al., 2023). A major concern with doping steel with toxic metals is that the metal ions released also harm humans and other life. Therefore, this approach is unlikely to be suitable for any structures exposed to soil or water resources or for water supply or medical applications.

Rare earth elements, such as cerium, are less toxic to humans than copper yet have antimicrobial activity (Wakabayashi et al., 2016). The inclusion of a limited quantity of rare earth elements has a relatively minor impact on the overall fabrication cost. Laboratory studies have demonstrated that incorporating cerium into steel can lessen microbial corrosion, but there is a dearth of practical assessments regarding the antibacterial efficacy of rare earth metals on authentic microbial corrosion communities (Jing et al., 2007; Yin et al., 2021; Yuan et al., 2013).

High‐entropy alloys are composed of multiple metals, such as iron, nickel, manganese, chromium, copper, or cobalt, in near‐equal proportions. They have improved mechanical properties such as weight/strength ratios (Gludovatz et al., 2014; Yang et al., 2023). High‐entropy alloys have antimicrobial properties that enhance resistance to microbial corrosion in laboratory studies (Chen et al., 2022; Gao, Jin, et al., 2022; Lei et al., 2022; Ren et al., 2022; Zhou et al., 2020; Zhou, Ren, et al., 2022). However, the high cost of nickel, chromium, copper, and cobalt makes high‐entropy alloys uneconomic for large‐scale applications, and the carcinogenicity of nickel and cobalt limits applications in medical devices. Non‐ferrous metals, most notably titanium, are more resistant to corrosion than ferrous metals, but titanium costs 30‐fold more than iron (Qiu & Guo, 2022), limiting applications to small‐scale devices.

### Non‐metallic coatings

Polymer coatings can prevent the formation of corrosive biofilms (Sethi et al., 2021; Zuo, 2007) and are employed in pipelines in the oil and gas industries (Guo et al., 2018; Kausar, 2018; Pranantyo et al., 2016). Polyurethane, polyimides, and silicone polymer coatings provide good protection at low cost (Brady, 1999; Guo et al., 2018; Ramezanzadeh et al., 2015). However, microbes can degrade the polymer coatings, reducing polymer adhesion (Ulaeto et al., 2023). The effectiveness of polymer coatings in preventing microbial corrosion can be improved with the incorporation of copper or silver to release toxic metal ions as the polymer is degraded, but this approach suffers from the limitations of toxic metal ions discussed above (Little et al., 2020). Quaternary ammonium salts can be covalently grafted onto polymer‐coated substrate surfaces. The exposed positively charged groups enable contact with the negatively charged microbial cell walls, inducing cell death (Huang et al., 2008; Klibanov, 2007; Tiller et al., 2001).

‘Smart coatings’ encapsulate antimicrobial substances or compounds that repair metal damage and respond to environmental stimuli (Udoh et al., 2022; Yu et al., 2023). For example, a metal‐organic framework (MOF) zeolitic imidazolate framework‐8 (ZIF‐8) preloaded with metronidazole or hollow mesoporous silica nanoparticles specifically responded to the activity of sulphate reducers, diminishing their corrosive activity (Cai et al., 2021; Tang et al., 2023). Zinc in the ZIF‐8 reacted with sulphide dissolving the ZIF‐8 and releasing the antimicrobials (Cai et al., 2021). A self‐healing, antimicrobial polymer effective in preventing microbial growth in laboratory studies while simultaneously healing damaged metal was fabricated with isophorone diisocyanate (IPDI) and the biocide 4,5‐dichloro‐2‐n‐octyl‐4‐isothiazolin‐3‐one (DCOIT) (Song et al., 2021). The IPDI released in scratched regions reacted with water to form a new polymer barrier to protect the metal surface, and the released DCOIT killed microbes. Evaluation of smart coatings has been limited to laboratory studies, and data on long‐term effectiveness and practical cost‐benefit analyses have not yet been reported.

## Is more sustainable ‘green’ corrosion mitigation possible with microbes?

The chemical production of biocides and coatings can be energy‐intensive, often involving toxic reactants and/or products and associated manufacturing waste. Microbial or plant‐based synthesis offers the possibility of being more energetically and environmentally sustainable. Less toxic products are conceivable with bio‐based approaches. Directed biological control or environmental manipulation to eliminate the most corrosive microbes seems feasible. These options are explored in the following subsections.

### Natural biocides, biosurfactants, and biofilm dispersants

Production of inhibitors of corrosion biofilm growth and activity with microbes or plants from renewable feedstocks may be more sustainable than the chemical synthesis of currently employed corrosion‐prevention biocides. Furthermore, biologically produced inhibitors with low human toxicity may expand the range of environments in which biocides can be deployed for corrosion prevention.

D‐Limonene, a common flavour enhancer in food preservatives derived from citrus plants (Sun, 2007), enhanced the biocidal impact of THPS on an oil field biofilm consortium in lab studies (Unsal et al., 2022). D‐amino acids, another natural organic product (Genchi, 2017), are signals for biofilm dispersal and can enhance the efficacy of commercial biocides like THPS and ADBAC (Jia, Yang, et al., 2017; Jia, Yang, et al., 2019). The cyclic peptide, Peptide A (CSVPYDYNWYSNWC), inspired by the natural equinatoxin II protein, enhanced the activity of the commercial biocide 2,2‐dibromo‐3‐nitrilopropionamide (DBNPA) against an oilfield biofilm consortium for two months (Wang et al., 2020) and the adhesion of a mixed marine microbial consortium (Herzberg et al., 2021). The antimicrobials indolicidin, bactenecin, and probactenecin secreted from genetically constructed *Bacillus subtilis* strains inhibited the growth of corrosive *Desulfovibrio vulgaris* and *Desulfovibrio gigas* on stainless steel (Jayaraman et al., 1999). Although organic biocide enhancers have shown promise in laboratory studies, field trials at large scale and under the complex conditions of ‘real‐world’ corrosion have yet to be conducted.

Biosurfactants, which can be produced with microorganisms or plants or synthesized from natural raw materials (Farias et al., 2021), are a potential alternative to biocides for preventing microbial corrosion (Płaza & Achal, 2020; Verma et al., 2023). Rhamnolipid, a glycolipid‐based biosurfactant produced by *Pseudomonas aeruginosa* (Nitschke et al., 2005), inhibited the corrosion of carbon steel by the corrosive microbe *Bacillus licheniformis* (Li, Yuan, et al., 2022). Adsorption of rhamnolipid on the metal surface yielded a physical barrier layer impeding biofilm formation (Li, Yuan, et al., 2022). An antimicrobial biosurfactant produced by a *Bacillus* species common in oil reservoirs adheres to metal surfaces, inhibiting biofilm adhesion and eradicating preexisting biofilms of a corrosive *Pseudomonas* species (Purwasena et al., 2019).

Quorum‐sensing inhibitors can interfere with the biofilm formation of some microbes and have been proposed as an additive to prevent microbial corrosion of metals (Grandclément et al., 2016; Lamin et al., 2022; Muhammad et al., 2020; Scarascia et al., 2016). Methyl eugenol, a plant‐produced quorum sensing inhibitor, disintegrated biofilms of corrosive *Desulfovibrio* sp. on stainless steel, limiting corrosion (Packiavathy et al., 2019). A mixture of quorum sensing inhibitors (Z‐)‐4‐bromo‐5‐(bromomethylene)‐3‐butyl‐2(5H)‐furanone, 3‐oxo‐C12‐(2‐aminocyclohexanone), and γ‐aminobutyric acid led to lower expression of *Desulfovibrio vulgaris* genes involved in anaerobic respiration, cell activity, biofilm formation, and corrosion of carbon steel (Scarascia et al., 2019). Quorum sensing inhibitors will need to be tested against complex microbial communities because of the likely specificity of any given quorum‐sensing inhibitor to a limited range of microbes (Jia, Unsal, et al., 2019).

As with biologically produced biocides, biosurfactants and quorum sensing inhibitors have yet to be evaluated in large scale trials. Production optimization and technoeconomic analyses will also be required before practical application in microbial corrosion mitigation can be realized.

### Microbes that protect metal surfaces

Employing microbes to thwart the activity of their corrosive cousins, or abiotic corrosion reactions, is an attractive potential alternative to the corrosion mitigation approaches described above, offering the possibility of robust, sustainable corrosion mitigation at low cost. Not all microbes promote corrosion, and biofilms or extracellular products can block O_2_ and microbial metabolites that attack Fe^0^ from contacting the metal surface. For example, marine *Vibrio* species form thick, homogenous biofilms on metal surfaces, serving as a physical barrier to metabolites and O_2_ (Gao et al., 2021; Gao, Zhang, et al., 2022). In addition to living cells, biomineralized membranes and extracellular polymeric substances (EPS) also protect the metal surface. A biomineralized film produced by *Pseudoalteromonas lipolytica* containing multiple layers of calcite and extracellular polymeric substances inhibited carbon steel corrosion (Guo et al., 2019). A thick biofilm of *Tenacibaculum mesophilum* D‐6 that formed on carbon steel surfaces consumed oxygen and inhibited the diffusion of corrosion species such as chloride ions (Li et al., 2021; Li et al., 2023). *Pseudomonas cichorii* and a *Vibrio* species deposited iron‐EPS complexes that inhibited steel corrosion (Chongdar et al., 2005; Moradi et al., 2015).

Developing a strategy to promote and maintain the growth of protective biofilms on metals in environments outside the laboratory that contain a diversity of microbes that will compete for the metal surface is likely to be challenging. However, there are models that might provide insights into how to accomplish this. For example, mineral deposits associated with the activity of *Methanobacterium* species were the apparent explanation for the low corrosion of iron sheet piles buried in marine sediments (in 't Zandt et al., 2019; Kip et al., 2017). Additional studies of this phenomenon and prospecting for other examples of microbial activity apparently slowing corrosion are warranted.

### Microbes that attack biofilm microbes

Phage therapy to rid environments of specific bacteria is a potential strategy to mitigate corrosion (Lou et al., 2021). Phage can selectively remove specific bacteria from complex microbial communities. For example, *Sphaerotilus natans* and *Haliscomenobacter hydrossis* are the primary causes of sludge bulking in wastewater systems. Introducing lytic bacteriophages that infect *S. natans* and *H. hydrossis*, isolated from mixed liquor samples of a wastewater treatment plant, reduced the numbers of these microbes and associated sludge bulking (Choi et al., 2011; Kotay et al., 2011). Two bacteriophages that specifically targeted *Salmonella enterica*, a main producer of hydrogen sulphide in sewage, significantly lowered *S. enterica* numbers and hydrogen sulphide production in synthetic sewage (Salim et al., 2021). Phages that diminished the growth of the corrosive oil pipeline microbe *Stenotrophomonas maltophilia* strain PBM‐IAUF‐2 were identified, but studies to determine the impact of the phage on corrosion were not reported (Pedramfar et al., 2017). Amendments of predatory *Bdellovibrio bacteriovorus* resulted in 80% less corrosion by sulphate reducers compared to controls (Qiu et al., 2016).

Clearly, there is limited information on the possibilities for employing bacteriophage and predatory microbes to control corrosion and biofilms. The effectiveness of such approaches within complex biofilms potentially comprised of a wide diversity of microbes is poorly understood. However, the strong appeal of such non‐toxic approaches to corrosion mitigation justifies further research.

### Modifying the environment to favour less corrosive microbes

The most severe corrosion is typically associated with the high activity of sulphate‐reducing bacteria because sulphide promotes the oxidation of Fe^0^ with the production of H_2_ (Little et al., 2020; Xu et al., 2023). Sulphate reduction is inhibited in the presence of nitrate because electron flow within microbial communities is diverted from sulphate reduction to the more thermodynamically favourable reduction of nitrate (Lou et al., 2021). Nitrate injection is a common strategy to prevent sulphate reduction in oil platforms and reservoirs (Okpala & Voordouw, 2018). Nitrate additions for 32 months reduced steel corrosion rates on a North Sea oil platform from 0.7 to 0.2 mm/y (Thorstenson et al., 2002). The number of sulphate‐reducing bacteria decreased 50‐fold. Nitrate amendments to another oil field reduced sulphate reducers 1000‐fold, with a 50% reduction in corrosion rate (Sunde et al., 2004).

## CONCLUSIONS

Many of the proposed possibilities for corrosion mitigation beyond the traditional approaches of scrapping, biocides, or substituting expensive metal alloys for cheaper structural iron have only been investigated in limited laboratory studies. Currently, publications of new mitigation strategies typically report their impact in the presence of a single species of corrosive microbe in incubations that are often no longer than a week. This is highly unrealistic. In environments in which corrosion is an economic concern, a broad diversity of microbes have months or years to initiate corrosion. Although it is more expensive and not always conducive to the time constraints of the students and postdoctoral students conducting academic research, longer corrosion tests are required. Studies need to be conducted under conditions that replicate the environments of concern, supporting the growth of diverse microbes that will have the opportunity to interact with metals in the real world.

Controlling the activity of diverse, complex microbial communities towards a desired goal is difficult. Therefore, the simplest path to preventing metal corrosion may be metallurgical rather than microbiological. It is already possible to fabricate ferrous metal alloys, like high‐quality stainless steel, that corrode much slower than more commonly employed steels. The issue with corrosion‐resistant alloys or metals like titanium is cost. Thus, technological innovations in alloy fabrication or metal mining will be necessary for a purely metallurgical approach to be economically viable.

Principles developed from decades of research on the bioremediation of contaminated environments may help guide the development of microbiological strategies for corrosion mitigation. For example, despite the academic lure of deploying genetically engineered microbes, this is probably not a practical alternative. Engineering microbes that release compounds that reduce the growth or biofilm formation of corrosive microbes is certainly doable, but less likely is that the engineered microbes will be able to survive and compete within the complex microbial communities associated with metal infrastructure and devices. A more practical legal concern is that most nations do not permit the broad‐scale release of genetically engineered microbes in open environments.

On occasion, introducing non‐genetically modified microbes selected for their bioremediation capabilities into contaminated environments can provide short‐term stimulation of contaminant remediation, but the probability for long‐term enhancement is limited without also modifying environmental conditions to promote the growth of the inoculum. Thus, prospecting for microbes that can prevent corrosion by forming protective layers of cells, exopolysaccharides, and/or mineral deposits may be a productive approach, but it will also require extensive research to identify how to modify environmental conditions to sustain these microbes against competition with more corrosive microbes.

The most economically viable approach to in situ bioremediation of contaminated environments is often a simple modification of environmental conditions to favour the growth of desired microbes. For example, the addition of electron donors to promote anaerobic dechlorination of chlorinated solvents (Xiao et al., 2020) or the reductive precipitation of contaminant metals (Williams et al., 2013) has proven effective, as has the addition of alternative electron acceptors to accelerate the anaerobic oxidation of hydrocarbon contaminants (Finneran & Lovley, 2003). Nitrate amendments to divert electron flow from sulphate reduction to nitrate reduction and diminishing sulphide production are analogous environmental manipulations to change the microbial community composition and activity to mitigate microbial corrosion. There may be other approaches that would be more cost‐effective and applicable to a broader range of environments. Further study of microbial communities, such as those apparently protecting marine sheet piles from corrosion (in 't Zandt et al., 2019; Kip et al., 2017), is required to better understand the optimum conditions to encourage their biofilm development and/or mineral deposition on metal surfaces. Soil and sediment conditions are likely to be much different than conditions within pipelines, or water resources, or the oral cavity, necessitating specific studies for each type of environment. The high economic burden of metal corrosion warrants such detailed investigations.

## AUTHOR CONTRIBUTIONS

**Di Wang:** Writing – original draft (equal); writing – review and editing (equal). **Enze Zhou:** Writing – original draft (equal); writing – review and editing (equal). **Dake Xu:** Writing – review and editing (equal). **Derek R. Lovley:** Writing – original draft (equal); writing – review and editing (equal).

## CONFLICT OF INTEREST STATEMENT

The authors declare no conflicts of interest.
